# Organizational politics and professional struggles in nursing

**DOI:** 10.1590/0034-7167-2022-0180

**Published:** 2023-03-06

**Authors:** Iraci do Carmo de França, Pacita Geovana Gama de Sousa Aperibense, Jane Márcia Progianti, Maria Angélica de Almeida Peres, Antonio José de Almeida, Tania Cristina Franco Santos

**Affiliations:** I Hospital Maternidade Carmela Dutra. Rio de Janeiro, Rio de Janeiro, Brazil; II Universidade Federal do Rio de Janeiro. Macaé, Rio de Janeiro, Brazil; III Universidade do Estado do Rio de Janeiro. Rio de Janeiro, Rio de Janeiro, Brazil; IV Universidade Federal do Rio de Janeiro. Rio de Janeiro, Rio de Janeiro, Brazil

**Keywords:** Nursing, History of Nursing, Professional Association, Organizational Policy, Societies, Nursing., Enfermeria, Historia de la Enfermeria, Asociaciones Profisionales, Política Organizacional, Sociedades de Enfermería., Enfermagem, História da Enfermagem, Associações Profissionais, Política Organizacional, Sociedades de Enfermagem.

## Abstract

**Objective:**

to analyze the professional struggles between nursing organizational entities, in Rio de Janeiro, during the Regional Nursing Council’s electoral process (1990-1993 administration).

**Method:**

historical study. We used journalistic articles, normative documents, legislation and semi-structured interviews with five nursing professionals who participated in this process. Interpretation of findings was supported by Bourdieu’s concepts of habitus, field, capital, and symbolic power.

**Results:**

Electoral Code changes of the aforementioned council, under the influence of administration (1987-1990), candidate for re-election, influenced the disclosure and eligibility criteria, making it difficult for broad participation, especially of Associação Brasileira de Enfermagem Rio de Janeiro Section.

**Final considerations:**

nursing, in this period, generated a field of disputes related to positions of power and gender, which was evidenced in the electoral process studied, which highlighted using limiting strategies by a group, making it difficult for the entire category to participate.

## RESUMO

**Objetivo:**

analisar as lutas profissionais entre entidades organizativas da enfermagem, no Rio de Janeiro, durante o processo eleitoral do Conselho Regional de Enfermagem (gestão 1990-1993).

**Método:**

estudo histórico. Utilizamos matérias jornalísticas, documentos normativos, legislações e entrevistas semiestruturadas com cinco profissionais de enfermagem que participaram desse processo. A interpretação dos achados foi apoiada pelos conceitos de *habitus*, campo, capital, e poder simbólico de Bourdieu.

**Resultados:**

as alterações no Código Eleitoral do citado conselho, sob influência da gestão (1987-1990), candidata à reeleição, influenciaram na divulgação e nos critérios de elegibilidade, dificultando a ampla participação, especialmente, da Associação Brasileira de Enfermagem Seção Rio de Janeiro.

**Considerações finais:**

a enfermagem, nesse período, gerou um campo de disputas relacionadas às posições de poder e de gênero, o que se evidenciou no processo eleitoral estudado, que ressaltou o uso de estratégias limitantes por parte de um grupo, dificultando a participação de toda a categoria.

## RESUMEN

**Objetivo:**

analizar las luchas profesionales entre las organizaciones de enfermería de Río de Janeiro, durante el proceso electoral del Consejo Regional de Enfermería (gestión 1990-1993).

**Método:**

estudio histórico. Se utilizaron artículos periodísticos, documentos normativos, legislación y entrevistas semiestructuradas con cinco profesionales de enfermería que participaron de este proceso. La interpretación de los hallazgos se apoyó en los conceptos de habitus, campo, capital, y poder simbólico de Bourdieu.

**Resultados:**

cambios en el Código Electoral del mencionado consejo, bajo la influencia de la gestión (1987-1990), candidato a la reelección, influyeron en los criterios de divulgación y elegibilidad, dificultando la amplia participación, especialmente de la Associação Brasileira de Enfermagem Sección Río de Janeiro.

**Consideraciones finales:**

la enfermería, en este período, generó un campo de disputas relacionadas con posiciones de poder y de género, lo que se evidenció en el proceso electoral estudiado, que destacó el uso de estrategias limitantes por parte de un grupo, dificultando la participación del conjunto la categoría.

## INTRODUCTION

The Federal Nursing Council (COFEn - *Conselho Federal de Enfermagem*) and the Regional Nursing Council (COREn - *Conselho Regional de Enfermagem*) were created in 1973 by virtue of Law 5905^(1)^. However, the first organizational entity of nursing was created in 1926, under the name of Brazilian National Association of Graduated Nurses (ANED - *Associação Nacional de Enfermeiras Diplomadas*), current Brazilian Association of Nursing (ABEn - *Associação Brasileira de Enfermagem*)^(2-4)^.

Thus, for almost 50 years, ABEn was the only entity responsible for the profession legal framework. Nevertheless, the struggles developed by the association for creating a supervisory entity for the profession have records since 1944, when important initiatives are evidenced regarding the study of four subjects of great importance for nursing at the time: supervision of professional practice; Nurses’ unions in the hands of nurses; nurses’ salaries; and the Public Service Administrative Department (DASP - *Departamento Administrativo do Serviço Público*) competition^(5)^.

ABEn’s concern with the creation of a council is evidenced and ratified over the subsequent decades. This need was recurrent for an official theme in several Brazilian nursing congresses, including forwarding the version of COFEn creation process to the National Congress^(5-6)^.

Despite being created in 1973, COFEn was only installed two years later. Thus, on April 23, 1975, the date chosen by the then president of ABEn, Maria da Graça Simões Corte Imperial (administration 1974-1976), with the Minister of Labor’s assent, Arnaldo Prieto, the COFEn board were sworn in at the Ministry itself. That same year, this board started work, successfully installing COREn in twenty states of the Federation^(1,7)^.

In the case of Rio de Janeiro, through COFEn Ordinance 1 - Rio de Janeiro, of August 4, 1975, a Special Board was established for creating a COREn in that city. This Board was sworn in and remained installed at the ABEn Section Rio de Janeiro (ABEn-RJ) headquarters ^5)^, at the time located on Avenida Franklin Roosevelt, in the center of the city.

Between 1975 and 1990, COREn in Rio de Janeiro (COREn-RJ) had five administrations. The first four were presided over by nursing professors from public institutions of higher education (*Universidade do Estado do Rio de Janeiro* and *Universidade Federal do Rio de Janeiro*) and linked to ABEn. The fifth administration (1987-1990) had a nurse working in a hospital as president, an unprecedented situation in the history of COREn-RJ until then.

In addition to significant performance in partnership with COREn-RJ, ABEn-RJ participated in all electoral processes of this supervisory body, offering political and social support to the entity, which represented the category union for professional interests. However, when the electoral process for the 1990-1993 COREn-RJ administration was about to take place, a change in its administration and organization was instituted, which ended up hindering the broad participation of professionals linked to nursing class entities (ABEn, Union of Nurses and Union of Nursing Assistants and Technicians). Thus, the following question arises: how did nursing organizational entities defend their positions in the face of this political field during the electoral process for COREn-RJ, 1990-1993?

The study is relevant due to the controversy surrounding the historical phenomenon, which involved the existence of denunciations about the aforementioned electoral process, reports in newspapers and large-circulation news programs, occurred on January 28, 2005, reporting Operation Predator, which aimed at combating crimes of embezzlement of public resources from the COFEn/COREn system, culminating in the arrest of COFEn’ arrest at the time and 14 other people. Understanding and reflecting on the historical facts of an electoral process in nursing allows expanding critical thinking, including on the current situation of Brazilian political-electoral processes, emphasizing the relevance of this study due to its micro-historiographical approach^(8)^.

The study is justified by the fundamental importance of historiography on the struggles for transparency in electoral processes, as they guarantee democracy and professional practice shaped by ethics. Therefore, it is extremely important that nursing has knowledge and awareness of its social role and the value of its work, in order to fight for dignity and respect, in dealing with its representation, in the sense of being informed of everything that comes to the agreement or disagreement of its rights and duties as a profession that generates science and social defense.

The organizational entities of nursing are the legal representations of nursing before society. The effective representativeness of these entails a greater interweaving and professional strengthening. The increase in research on this topic can contribute to greater attention by professionals to the profession’s political aspects and social commitment.

## OBJECTIVE

To analyze the professional struggles between nursing organizations in Rio de Janeiro, during the COREn electoral process (1990-1993 administration).

## METHOD

### Ethical aspects

This article comes from an academic master’s dissertation, defended in 2019, in which ABEn-RJ’s strategies were discussed in the struggle for nursing participation in the COREn electoral process in Rio de Janeiro, during the period of electoral processes for the 1990-1993 and 1993-1996 administrations. With regard to oral sources, in order to respond to the objective presented in this study, excerpts from five interviews were used, out of seven, used when developing the aforementioned dissertation. This research was approved by the Research Ethics Committee of the institution to which it was linked. Therefore, the study met all the formal requirements contained in Resolutions 466/12 and 510/2016 of the Brazilian National Health Council.

### Study design

This is a historical-social research, with a qualitative approach, as it is the study of a historical fact that prioritizes, as a field of interest, clippings of human relations or human clippings in a given context^(9)^. Furthermore, as it is a circumscribed study in the micro-history approach, the object of investigation is situated in a specific occurrence, i.e., a certain electoral process, considered to reveal broader professional problems, involving the professional organizations. This type of approach, by working with the reduction of observation scale, allows the apprehension of details that sometimes go unnoticed by the traditional macro-history^(8)^. The temporal cut encompasses the period from 1989 to 1991, whose first milestone refers to the outbreak of COREn-RJ’s electoral process for the 1990-1993 administration, and the end refers to development of this same process after inaugurating the elected administration.

### Methodological procedures

#### Study setting

The study setting corresponds to the space of power in which the electoral process for COREn-RJ’s 1990-1993 administration was triggered, which reflects an environment where symbolic struggles were established. It directly involves the COREn-RJ and COFEn headquarters as well as National ABEn and ABEn-RJ.

#### Theoretical-methodological framework

Discussion was carried out in light of French sociologist Pierre Bourdieu’s theoretical concepts, including habitus, field, capital and symbolic power. The concepts of habitus and field are central categories in Pierre Bourdieu’s explanatory theory of social world. The first is defined by Bourdieu as acquired knowledge, which is incorporated in the form of permanent dispositions that generate perceptions, appreciations and practices. The concept of field is a multidimensional space where the weight and volume of owned capital will determine the occupation of positions of power by different social actors. In this space, fights are engendered, which bring embedded symbolic violence, for the perpetuation of prestigious positions in these spaces. It is worth noting that symbolic power is exercised with the complicity of those who do not realize or do not want to realize that they are subject to^(10)^.

#### Data source

The findings come from direct historical sources, including written and oral documents. The writings are publications in DOE-RJ (Official Gazette of the State of Rio de Janeiro) on the electoral process dissemination, COFEn Resolution 52/1979, which defines COFEn Regulations, COFEn Resolutions 87/1986 and 117/1989, which define the Electoral Codes, and a letter addressed to nursing professionals, entitled “Coup in Nursing”, signed by several entities.

It is noteworthy that, in this technique, saturation criterion is not used, as the collaborator’s experience and point of view on the historical phenomenon are valued, which can be unique for each person^(11-12)^.

#### Data collection and organization

Written documentary sources were located at ABEn-RJ headquarters, at the Official Press of the State of Rio de Janeiro (*Imprensa Oficial do Estado do Rio de Janeiro*) and at the COFEn Library, in Brasília. We included documents pertaining to the time frame and the subject under study. We excluded documents that were erased or found torn, incomplete or whose reading was compromised.

Oral documents for developing the dissertation that gave rise to the present study were produced using the Thematic Oral History technique^(9,11-12)^, through semi-structured interviews with professionals directly or indirectly involved with the struggle strategies developed by ABEn-RJ, the Union of Nurses of Rio de Janeiro (SindEnfRJ - *Sindicato dos Enfermeiros do Rio de Janeiro*) and the Union of Nursing Assistants and Technicians of Rio de Janeiro (SATEMRJ - *Sindicato dos Auxiliares e Técnicos de Enfermagem do Rio de Janeiro*), six nurses and one nursing assistant. There was no withdrawal or refusal of any of them. Collaborators were identified with the letter “C” followed by the order in which the interviews were carried out, arranged from C1 to C7. The interviews were collected by one of the authors of the study, who was a master’s student at the time, between March and July 2017 and had an average duration of 56 minutes. Also, the researcher performed transcription and provided validity by collaborators after reading and agreeing with its content.

The data were worked on in a thematic analysis framework that facilitated understanding of its breadth and depth. The documentary corpus composition considered document relevance, sufficiency, completeness, representativeness, homogeneity and organization. Result reliability was achieved through the so-called external and internal reviews^(9)^, which analyze the document origin and the evidence contained therein. The documentary mass was considered as a whole and in articulation with the historical context of its production. In addition, the chronology of events during the period studied was considered.

#### Data analysis

From historical context triangulation, social structures and discourses identified in the written and oral documents, based on the theoretical framework^(13)^, explanatory texts were prepared through context unit analysis, in order to overcome the mere textual description, placing the social event (electoral process), in the social context, for constructing marked and consistent analyzes. This analysis made it possible to identify struggle strategies developed by ABEn-RJ, throughout the electoral process of COREn-RJ’s 1990-1993 administration, which was the chosen analysis category for the study.

## RESULTS

### Brazilian Association of Nursing: analysis of the 1990-1993 administration electoral process of the Regional Nursing Council of Rio de Janeiro

The study’s contextual background covers the turn of the 1980s, a period that corresponds to the return of democratic regime to Brazil after 21 years of military dictatorship (1964-1985), with the election of Fernando Collor de Melo, sworn in in March 1990^(14)^. It is in this context of political reopening, the return of social movements for guaranteeing political and social rights and the alleged improvement of quality of life, mainly in health and education, that nursing professionals resume their participation in broader issues of society and, particularly, issues of nursing and class entities, especially ABEn, which was also undergoing transformations^(15)^.

The neophyte COREn-RJ, since 1975, had ABEn-RJ’s leading role, mainly in the succession process. The first four COREn-RJ administrations were composed of prestigious nurses in higher education in nursing, and ABEn-RJ, since 1926, brought experience and social respect in the representation of nursing category.

The electoral process of the 1987-1990 administration, which elected Gilberto Linhares Teixeira, took place amidst a political opening movement in the country, started in the process of liberalization of military dictatorship, in 1974, culminating in 1988, with the promulgation of the new constitution. This feeling of change also occurred in nursing, which reverberated in the unprecedented formation of a competing list for COREn-RJ, formed predominantly by male professionals, not from educational institutions. This fact represents a major historical change in Brazilian nursing, as it marks the entrance and leadership of men in a space dedicated to the supervision of professional practice, in a profession that is mostly female. Excerpts from the speeches of some collaborators show the repercussions of the country’s political moment in nursing:

*The category was no longer satisfied* [...] *and the entities did not feel well represented* [...]. *It was when an opposition electoral alliance emerged, in the sense of being able to democratize for the category to have an effective participation.* (C2)

*As there was dissatisfaction* [with the current board], *the electoral alliance merely made up of men was not something that made the category surprise. The category thought the following, “They are men, but they are nurses linked to the service who know better than the academy”.* (C4)

It should be reported that the electoral process regimentally determined by Resolution is approved by COFEn plenary. The Electoral Code itself is defined based on the law that instituted the creation of Nursing Councils (5.905/73), which is responsible for “making provisions and issuing instructions for procedure uniformity and the proper functioning of COREn; “convene and hold elections for its board of directors”^(1)^.

The creation of Resolution that determines the Electoral Code is also based on the Autarchy Regulation, COFEn Resolution 52/1979 52/1979^(13)^, Article 16, Items XXI and XXII. COFEn Resolution 87 of December 18, 1986, which determines the councils’ Electoral Code, explains that the electoral process must be disclosed in the form of three notices (Electoral Notices 1, 2 and 3)^(16)^.

Electoral Announcement 1, based on COFEn Resolution 87/86, determines the call of General Assembly (meeting of nurses who work in the area under COREn’s jurisdiction, which is responsible for electing autarchy councilors), with election date, which must take place at least 130 days before the date set for election (in this Electoral Code), formalizing the opening of the electoral process and disclosing the electoral registration period alliances. In the said public notice, place, time and deadline indication for receiving the registration requirements of electoral alliances must also be included^(16)^.

Electoral Notice 2 must disclose, immediately after the deadline for the presentation of electoral alliances, communication that, at the council headquarters, the list of registered electoral alliances is available to interested parties, whose registration was requested as well as to determine the deadline, place and time for receiving objections^(16)^.

Electoral Notice 3 again publishes election date, places and times for voting and electoral alliances ratified by COREn plenary, with the nominal list of all components^(16)^.

The electoral process for the 1990-1993 administration was governed by three legal documents, such as the Resolution that conducted the previous electoral process (1987-1990 administration). By comparing them, two significant changes were identified in terms of disclosure and eligibility criteria. There was a restriction on the scope of disclosure, by removing the sole paragraph of Article 8, which determined publication in the Official Gazette and in all means available to the council. In its place, nothing was inserted, leaving the official publication restricted only to the Official Gazette. On eligibility, Article 18 includes the need for the candidate to declare that he or she was not a member of administration, administration or collegiate body (including tax) of a class entity, at any level.

Despite these changes, COFEn Resolution 117/89 contains references to the previous Electoral Code, respectively: “the Electoral Code provisions here remain in force, explicitly or implicitly, not amended or revoked” and “the provisions to the contrary are revoked”^(17)^. Regarding Electoral Code changes, one of the collaborators mentions that such changes can be interpreted as a strategy to make it impossible the participation of professionals who were not directly involved in the council’s routine, such as assistance and teaching professionals in general and those who were active in other entities, such as ABEn and unions. One collaborator thus commented on the fact:

*It created a device exactly for when it came to the electoral process* [1990-1993] *entities had difficulty composing electoral alliances.* (C2)

Thus, the employees add that this strategy aimed to maintain positions of power in the space of COREn-RJ. Participant recalls the introduction of Electoral Code’s Item VI in Article 7, as highlighted below:

*He* [COREn-RJ president] *already demonstrated that he was carrying out a policy with the intention of remaining in power, making another electoral process more difficult.* [...] *and the composition of opposition electoral alliances to run for the COREn/RJ election, preventing representatives of union entities from running for office.* (C2)

*It was a project of “empowerment”, not empowerment, but “empowerment” and expropriation of representation of nursing.* (C1)

Excerpts from the interviews deal with the mismatch between the administration (1987-1990) proposals, on the occasion of its electoral campaign, which emphasized better communication with the category and the restriction of disclosure of election to successor administration, legitimized by changes in Electoral Code:

*Dissatisfaction with the poor service given to the incumbents made the opposition candidate’s proposal the prospect of change. This happened at first: it started to modernize, improve service, computerize the council,* [...] *and, with that, he had been making a policy, demonstrating that it would be a new council, different from what the category knew.* (C2)

*It was board practice to manipulate information. So, the group that was able to enroll in the process within the established deadline was the group that internally had the privilege of being aware of the period in which the call notice would be published. So, they prepared their paperwork in a timely manner, while those on the outside who had every interest did not have enough time to prepare the required paperwork.* (C6)

The statements made by collaborators regarding Electoral Code changes of Nursing Councils are restrained in Article 29 of COFEn Resolution 117/89, which determined the immediate publication of the entire content of Nursing Councils’ Electoral Code and established a period of two weeks so that there was enough time for leaders to assimilate significant changes in text content (COFEn, 1989).

Considering that the publication date of COREn Resolution 117/89 was December 11, 1989, Public Notice 1 of the electoral process for the 1990-1993 administration could not have been published before December 26, 1989; however, the publication took place on December 22, 1989, with an interval of only 11 days, therefore, contrary to the Electoral Code determination^(18)^.

Excerpts from collaborators’ speeches show the difficulty of nursing in accessing information regarding the electoral process outbreak for the 1990-1993 administration:

*The Notice was published in the Official Gazette. It was not published in the Council Bulletin or in a widely circulated newspaper, so we, the entities, were left to do research, when the Public Notice was going to be published, a very short period, exiguous to compete. A series of notary documents, which at that time took* [...] *so all of this was a way to make it difficult.* (C2)

*At that moment* [90/93 administration] […] *he preached that he was going to expand nursing administration within the council, and he closed the council in a group where you did not have access to the accounts, which was a campaign promise, but which was never presented in detail in those council accounts.* (C4)

Electoral Notice 2, published on January 30, 1990, informs about the registration of one electoral alliance in Board I and two electoral alliances in Boards II and III. In Electoral Notice 3 of April 3, 1990, the following approvals are given: Electoral alliance 1 for Board I and Electoral alliance 1 for Boards II and III. Board II and III Electoral Alliance 2 was not approved. Certainly, the non-homologation occurred due to the impossibility of meeting all the documentary requirements for electoral alliance registration. The excerpt extracted from one of the interviews records such difficulties and the measures to overcome them:

*Our* [SATEMRJ] *legal department filed a lawsuit for us to run in that 1990-93 election through a preliminary injunction. We had a lot of difficulties getting the documentation that that resolution required. The Union of Nurses also encountered this great difficulty* [...] *there were many, many, many certificates.* (C2)

The injunction referred to by the collaborator is verified in the publication in DOE - RJ, on May 25, 1990, with the name “Term of Amendment to Electoral Notice 3”, determining the consideration of registration of electoral alliance 2 for Board I and electoral alliance 2 for Boards II and III, to run for election. The injunction favorable to electoral alliance 2 for Board I and Board II and III was released on May 25, 1990, and the voting day was kept the same, June 1, 1990, allowing only 5 days of campaigning for these electoral alliances. The results of the ballot boxes determined the victory of electoral alliance 1 of Board I and, surprisingly (considering the time they had to carry out the campaign), the victory for electoral alliance 2 of Boards II and III.

On October 31, 1990, COREn-RJ published in the DOE the result of the electoral process, based on COFEn plenary held on 30.10.1990. This date corresponds to the last day of the 1987-1990 administration of COREn-RJ^(19)^. Regarding election result consequences, two collaborators comment on tensions experienced by directors of Boards II and III in their daily work:

*It was a conflictual plenary, because it was a Board I situation, the majority within the plenary,* [...], *with the minority of Boards II and III* [...]. *We were outvoted there at the time, but we were in opposition even there, we started to denounce the practices that were put in place.* (C2)

*We left because they were able to revoke the injunction* [...] *the injunction was revoked today. The next day, he called a plenary and dismissed, placing the electoral alliance that received less than 2% which was the electoral alliance that articulated politically with Board I* [...] *and there this electoral alliance remained illegitimately.* (C3)

The lawsuit’s outcome was communicated and published in the DOE on May 8, 1991, by COREn-RJ, nullifying the injunction granted and, insubstantial, the provisional possession of professionals from Boards II and III^(20)^. That same communiqué also convened the new effective and alternate directors to compose the plenary. The nominal list of councilors shows that it includes professionals who made up electoral alliance 1 and who lost significantly in the votes. It also draws attention to the urgency of the summons to take office, determined for the same day of publication in the official gazette.

Members of electoral alliance 2 and several Health Unions released a letter, addressed to their professionals, entitled “Coup in Nursing”. In the letter, they inform that the dismissal of electoral alliance 2, called “Action and Equality”, elected with 85% of votes counted in election of June 1, 1990, outside a “coup” by the then president of COFEn, Gilberto Linhares.

Thus, it was recurrent the interviewees’ statement that the promised democratization of COREn-RJ did not materialize. In this sense, the collaborators added that the necessary disclosure of information by the municipality regarding the electoral process did not happen as expected.

## DISCUSSION

COFEn/COREn system Electoral Code changes made it difficult for the boards of professional associations to participate in competition for election. This is an unprecedented situation in the historical trajectory of autarchy’s electoral processes, which always had the participation of recognized nurses, especially in nursing education, and who occupied important positions, especially with ABEn partnership, where partner nurses acted as full-time board members. This assertion, as we have seen, was supported by the statement of an interviewee, when he stated that Electoral Code changes symbolized, in his perception, the intention of perpetuating power by those who occupied the 1987-1990 administration at COREn-RJ, as well as the impossibility of competing in election by nursing professionals linked to other political conjunctures, as is common in democratic processes, which should allow free choice by voters of the slate that best meets the needs of the majority of the group involved. Certainly, the symbolic capital of these professionals, raised in and by nursing, had an important weight in attracting votes for electoral alliance, representing a threat to opposition electoral alliance.

Relating these facts to the validity of a nursing standard in Brazil (1931-1949), the diffusion of a professional nurse identity in society, initially female and formed with a distinct profile, built on the formation of a boarding school disciplinary regime and high academic demands, it had an impact on the social division of work in the category, which came to be headed by this group. The 18-year period (1931-1949) in which the standard lasted formed a nursing leadership, whose professional habitus, in the following decades, faced some opposition, resulting in a separation that presents itself as the field of education and the field of nursing practice, which is perceived even when organizing an electoral alliance to compete for professional entities.

The release of Public Notice 1, on December 22, 1989, Friday, the day before a world holiday (December 25), had important implications, since, with the recess of the Federal Court, the issuance of the necessary certificates for qualification to enroll electoral alliances was significantly impaired. Moreover, as it was a period close to the Christmas holiday, many professionals were on vacation or on break for the end of the year festivities. The publication of this Notice took place exactly 11 days after the publication of COFEn Resolution 117/89, published on December 11, 1989. The time span between these publications does not comply with Article 29 of COFEn Resolution 117/89, which establishes a minimum of 15 days^(18)^. This fact was ignored, which allows us to analyze it as one more strategy to suppress the participation of new electoral alliances.

In order to guarantee the registration of opposition electoral alliances in the electoral process, it was necessary to judge the process. As polling day was maintained, said electoral alliances had only two business days to campaign. Despite the short time, electoral alliance 2 of Boards II and III won the election.

In an electoral process, the voting group’s voice is heard by the votes deposited in the ballot box. Thus, nursing in Rio emphatically expressed itself in favor of an electoral alliance that, at that moment, did not unite with the ideas of a group that already participated in the council and that was willing to remain with its political project. There is legitimacy in direct elections, and the vote has decision-making power, because it is the majority’ choice and must be respected.

With regard to composition of elected electoral alliance of Board I, of its 24 members, 19 were male, which corresponds to 79.1%, a percentage inversely proportional to the category’s representativeness of approximately 80% of female professionals as well as in the composition of the first four COREn-RJ administrations. Female participation in politics must be discussed in the light of gender references, and scholars on the subject show that, in Brazil, a historical issue still leaves women out of political spaces for reasons that involve, mainly, a supposed intellectual supremacy and male workforce^(21)^.

Since the beginning of the 20^th^ century, Brazilian nursing has supported a female elite to assume leadership positions in the professional space, composed of well-prepared professionals, that is, teaching nurses with greater academic training. This strategy was a way to increase these women’s conditions of confrontation in the political-social field. ABEn, as the oldest nursing entity, brings this reality into its trajectory, which, above all, represented nursing as a profession with a female majority. The social condition of women requires a look at gender specificities that are not fully respected in the labor market, such as wage equality and professional advancement^(22)^, which leads to strangeness when there is a group that wants to represent nursing without a female representation equivalent to its quantitative in relation to gender.

It is worth mentioning that the men who made up electoral alliance 1 of Board I came from assistance in hospital institutions, which certainly made it possible for them to rise to power, since, in these spaces, occupied mostly by physicians and men, the reproduction of male power over nurses is more efficient, i.e., the volume and weight of professional capital of men (physicians and nurses) exert greater power of domination. It should be emphasized that consecrated nurses, i.e., holders of symbolic capital to occupy positions of command, for the most part, demarcated their successful professional trajectory in educational institutions and class entities.

The strong male presence in the autarchy of a mostly female profession, as well as the professional capital of the winning electoral alliance components, demonstrates a new social order, in which the weight and volume of nurses’ symbolic capital were not enough to secure ABEn’s positions of power in the command of COREn-RJ (1990-1993 administration).

The strategies developed by COREn-RJ presidency (1987-1990 administration) regarding changes to the electoral process legal provisions for the following administration (1990-1993) aimed at maintaining the group in command of the council. Such strategies, which aim to make or break groups, combined with the collective actions that these groups can develop to transform the social world according to their interests, made the perpetuation of power possible. Through the power institutionalized by administration, heretical ruptures of this established order were made difficult.

Nevertheless, the symbolic struggles developed by class entities and by the opposition electoral alliance contributed to publicly professing the rupture with the new established order, because “heretical subversion explores the possibility of changing the social world by modifying the representation of that world”^(23)^. A historical study on the articulation of nursing representative entities and their influence on the profession demonstrates that creating the council provided a first period of disarticulation and fragmentation that directly influenced the profession valuation before society and the confidence of professionals themselves in relation to their representation bodies^(24)^. On the other hand, an integrative review identified that nursing entities are important and necessary, as they have contributed decisively to the struggles of the category in favor of the class and society in general with regard to the various axes of training, performance and professional qualification consistent with the class’s and social demands’ interests^(25)^.

The resistance strategies developed by the entities in the 1990-1993 administration electoral process were possible, through the understanding that “the social order partly owes its permanence to the imposition of classification schemes which, by adjusting to objective classifications, end up producing a form of recognition of this order”^(23)^. In this way, recognizing the lack of knowledge of arbitrariness of social order foundations contributed to carrying out strategies to break compliance with the established social order.

Collaborators’ statements show that the opposition group faced a lot of resistance, which was materialized by strategies to jettison the group even by the use of space on the council premises. The urgency of summoning the new councilors to take office, the day after announcing injunction cancellation, led electoral alliance II to use the strategy of sending an open letter to nursing in Rio de Janeiro, denouncing the events. This letter was signed by members of Unions of Workers in Health and Social Security, SATEMRJ, SindEnfRJ, National Federation of Nurses, Union of Physicians, Union of Psychologists, Union of Speech Therapists, Union of Health Workers of South Rio de Janeiro and Union of Health Workers of the State of Rio de Janeiro, in addition to ABEn.

The support expressed by the aforementioned institutions gives visibility and recognition to electoral alliance II, as they are institutions endowed with political capital, which functioned as symbolic weapons in the struggle for the demand that the democracy principle be exercised in the ongoing electoral process.

In opposition to the situation group, there were national leaders of nursing, prestigious and respected for their actions in higher education institutions, which were strongly linked to ABEn, since its creation, in 1926. In fact, the association was born on the premises of a school of nursing: *Escola de Enfermagem Anna Nery* ^(26)^. This group certainly constituted a threat to the established order, as they were professionals linked to nationally recognized institutions.

### Study limitations

Some documents produced by the administration, which took over the period studied, were lost, making it impossible for researchers to access them, which could have expanded the analysis and discussion of findings. About the oral sources, it is recorded that their production is based on experiences and emotions of an individual nature, rescued according to the present’s memory, experiences and needs, therefore, limited by memory that study collaborators have of the facts that occurred.

### Contributions to nursing, health, or public policies

The study contributes to deepening the historiography of nursing’s organizational entities, with an unprecedented thematic cut that brings to light an erudite historical version and buoyed by documents about part of the association’s fight for transparency in electoral processes, in addition to highlighting ABEn’s role in political defenses to strengthen the profession and social control, strengthening democracy and professional practice shaped by ethics.

## FINAL CONSIDERATIONS

The electoral process for the 1990-1993 administration was conducted by the plenary of councilors who took office in the previous election: the 1987-1990 administration, when Gilberto Linhares becomes president of COREn-RJ for the first time. Thus, Electoral Code changes were influenced and facilitated by occupation of an important position of power which, in turn, made it possible to obtain more power in the space under study. Electoral Code changes made it difficult for nursing to participate in articulation with professional associations in this electoral process, culminating in its non-representativeness in administration, leading to a break in relations.

By creating symbolic struggle strategies to make the electoral process of COREn-RJ, for the 1990-1993 administration, accessible to the entire nursing community, ABEn-RJ relied on essential allies, such as SindEnfRJ and SATEMRJ.
